# Probabilistic risk assessment and spatial distribution of potentially toxic elements in groundwater sources in Southwestern Nigeria

**DOI:** 10.1038/s41598-019-52325-z

**Published:** 2019-11-04

**Authors:** PraiseGod Chidozie Emenike, Imokhai Tenebe, Nkpa Ogarekpe, David Omole, Chidozie Nnaji

**Affiliations:** 1 Department of Civil Engineering, Covenant University, Ota, Ogun State Nigeria; 2 Ingram School of Engineering, Ingram School of Engineering, Texas State University, San Marcos, Texas USA; 3 Department of Civil Engineering, Cross River University of Technology, Calabar, Nigeria; 4 Department of Civil Engineering, University of Nigeria, Nsukka, Enugu State Nigeria; 5 Faculty of Engineering and Built Environment University of Johannesburg, Johannesburg, South Africa

**Keywords:** Environmental monitoring, Environmental impact

## Abstract

The study investigated the concentration of potentially toxic heavy metals (PTHM) in groundwater sources (hand-dug wells and boreholes), spatial distribution, source apportionment, and health risk impact on local inhabitants in Ogun state. One hundred and eight water samples from 36 locations were analysed for Cr, Ni, Pb, Fe, Mn, Mg, Ca and Al. Mean values of 0.013, 0.003, 0.010, 0.088, 0.004 and 3.906 mg/L were obtained for Pb, Cr, Ni, Fe, Mn, and Al respectively at Iju district. Meanwhile, the average values of Pb, Ni, Fe, Mn, and Al concentrations at Atan district were 0.008, 0.0023, 0.011, 0.003, and 1.319 mg/L respectively. Results also revealed that the 44.4% and 11.13% of the borehole and well-water samples surpassed the World Health Organization limits for Pb at Atan. In Iju, the concentration of Pb and Al were relatively high, exceeding the stipulated standard in 100% of the samples. The Multivariate statistical analysis employed produced principal factors that accounted for 78.674% and 86.753% of the variance at Atan and Iju region respectively. Based on this, PTHM were traced to geogenic sources (weathering, dissolution, leaching) and anthropogenic emissions from industrial activities. In addition, the hazard quotient values obtained from the health risk assessment identified potential non-carcinogenic risk due to Pb via ingestion route. Ni was found to have high carcinogenic risk on adult and children, having exceeded the threshold limit. The outcome of the carcinogenic risk assessment revealed that 88.67% (for adults) and 1.12% (for children) of the cancer risk values surpassed the specified limits at Iju, whereas the cancer risk values were considerably lesser at Atan. In conclusion, the report of this study should serve as a beacon that will spark up strategic planning, comprehensive water resource management, and extensive treatment schemes in order to address the health complications linked with environmental pollution.

## Introduction

Global attention has been directed towards environmental deterioration due to the threat it poses to living creatures and standard of living^1,2^. Agricultural activities, industrialisation and urbanisation have emerged as a prime contributor to the rising environmental pollution affecting human lives^3–5^. Observing this, groundwater, which serves as a drinking source in most countries, continue to be polluted with toxic contaminants such as heavy metals (HMs)^6,7^. According to Fallahzadeh *et al*.^8^ and Khamirchi *et al*.^9^, poor waste management of waste resulting from industrial and allied process is largely responsible for groundwater contamination observed around the world. Hadley and Newell,^10^ and Chabukdhara *et al*.^11^ added that the indiscriminate release of industrial waste into the environment could lead to a rise in groundwater contamination.

HMs are classified as toxic because they can penetrate water aquifers and consequently, agglomerate in the food chain^12–14^. It is important to mention that heavy metal toxicity is based on the fact that they are non-biodegradable, persistent, xenobiotic and can accumulate in humans over a time^15–18^. HMs such as chromium (Cr). Copper (Cu) and zinc (Zn) are considered toxic to biota when their thresholds are exceeded^19^. Arsenic (As) can cause visceral cancer^20^ as well as liver, and bladder cancer^21^. Cadmium (Cd) can cause a decline in cognitive capacity, renal disorder and consequently, bone loss^22–24^. Lead (Pb) affects the central nervous system (CNS), especially in children, thereby causing hyperactivity, fatigue, anaemia and diminished IQ^25–28^. Furthermore, the nutritional demand of HMs varies considerably between different species and the gap between their optimum concentration are quite low. Thus, exposure to an excess concentration of heavy metal proportions may lead to severe nervous imbalance and possibly death^29^

Considering the adverse health effect and possible death threat posed by HMs on living organism, constant exposure to polluted groundwater sources poses a serious risk to human health. Shankar *et al*.^30^ reported that groundwater contamination had adverse effect on almost 14% of the global population. Hence, it is essential to monitor the quality of water consumed in order to safeguard human health, especially those dwelling around industrial regions^7^. Besides, it is crucial to conduct health risk assessment (HRA) for dwellers considering various exposure pathways in line with the recommendations of US EPA^31^. US EPA generated HRA for carcinogens and non-carcinogens after considering the report of the International Agency for Research on Cancer (IARC) relating to the carcinogenic potentials of chemical pesticides^31,32^, and it has been verified to be a vital and effective tool used for water pollution studies^11,33–36^.

Recently, researchers have adopted Monte Carlo Simulation (MCS) technique for the evaluation of hazard potential and pollution risk in different environments^8,11,20,37–40^. MCS quantifies variability and caters for the uncertainties associated with risk assessments^41–43^. The technique works by quantitative estimation of the probability distribution for exposure and health risk analysis^44^. Also, the inclusion of geostatistics to obtain the spatial distribution of contaminants, observe environmental conditions, explain scientific results, and proffer water management solutions have been adopted by several researchers^45–50^. To handle large datasets containing diverse variables, investigators have adopted the use of statistical tools to transform the datasets into factual interpretations useful for general conclusions^15,33,49,51–53^

Urban regions in Nigeria have experienced water fluctuations that resulted in the exploitation of groundwater resources (hand-dug wells and boreholes) for industrial, domestic and agricultural purposes. Due to industrialisation and urbanisation, there has been an increase in human activities and the situation has resulted in further pollution of groundwater sources. Major industries around Ado-Odo Ota, Ogun State, Nigeria have exposed dwellers to chronic health conditions traceable to consumption of contaminated groundwater. Kayode *et al*.^54^ conducted water analysis in neighbouring towns of Ifo, Agbara and Abeokuta and heavy metals and reported a preponderance of heavy metals in the water samples investigated. Other investigators that carried out water analysis within Ado-Odo Ota discovered a similar case^55,56^. However, up till now, there is lack of regional health risk studies despite the growing population within the region engendered by the availability of land, job opportunities (due to the presence of industries), and available market. The stress exerted on water distribution systems have become unbearable due to the rising water demand within the region. Therefore dwellers have resolved to over-exploitation of groundwater reservoirs^57^.

Recently, Emenike *et al*.^17^ reported the HRA of sachet water sold within Ado-Odo Ota. Meanwhile, Tenebe *et al*.^58^ reported HRA of surface water within Ado-Odo Ota. However, to the best of our knowledge, there is insufficient literature of previous studies regarding HRA of boreholes and hand-dug wells within the region. Therefore, the objectives of this study focus on the following. First, to evaluate the concentration of HMs in boreholes and hand-dug wells. Second, to investigate the non-carcinogenic and lifetime cancer risk associated with HMs. Third, to determine the spatial distribution of HMs by adopting geospatial technique. Finally, to predict the HMs sources with the aid of statistical tools. Unfortunately, there are no derived guidelines for evaluating risk assessment of contaminants in Nigeria and Africa at large and very few works have considered the use of probabilistic risk assessment on environmental quality indicators within the region. Therefore, it is imperative to state that this study might be the first to conduct a probabilistic estimation of risk exposure within the study region using MCS technique. In addition, Iju and Atan harbours several farmlands and small-medium-enterprises that plays a significant role in the socio-economic advancement of the region. Also, close proximity to the Benin Republic border makes the region an attractive location where industries are located. Therefore, our aim is to ensure that ecotoxicology assessments are included in monitoring schemes—an aspect yet to receive recognition when correspondence relating to contamination are examined in Nigeria.

## Materials and Method

### Study area

**Figure 1:**
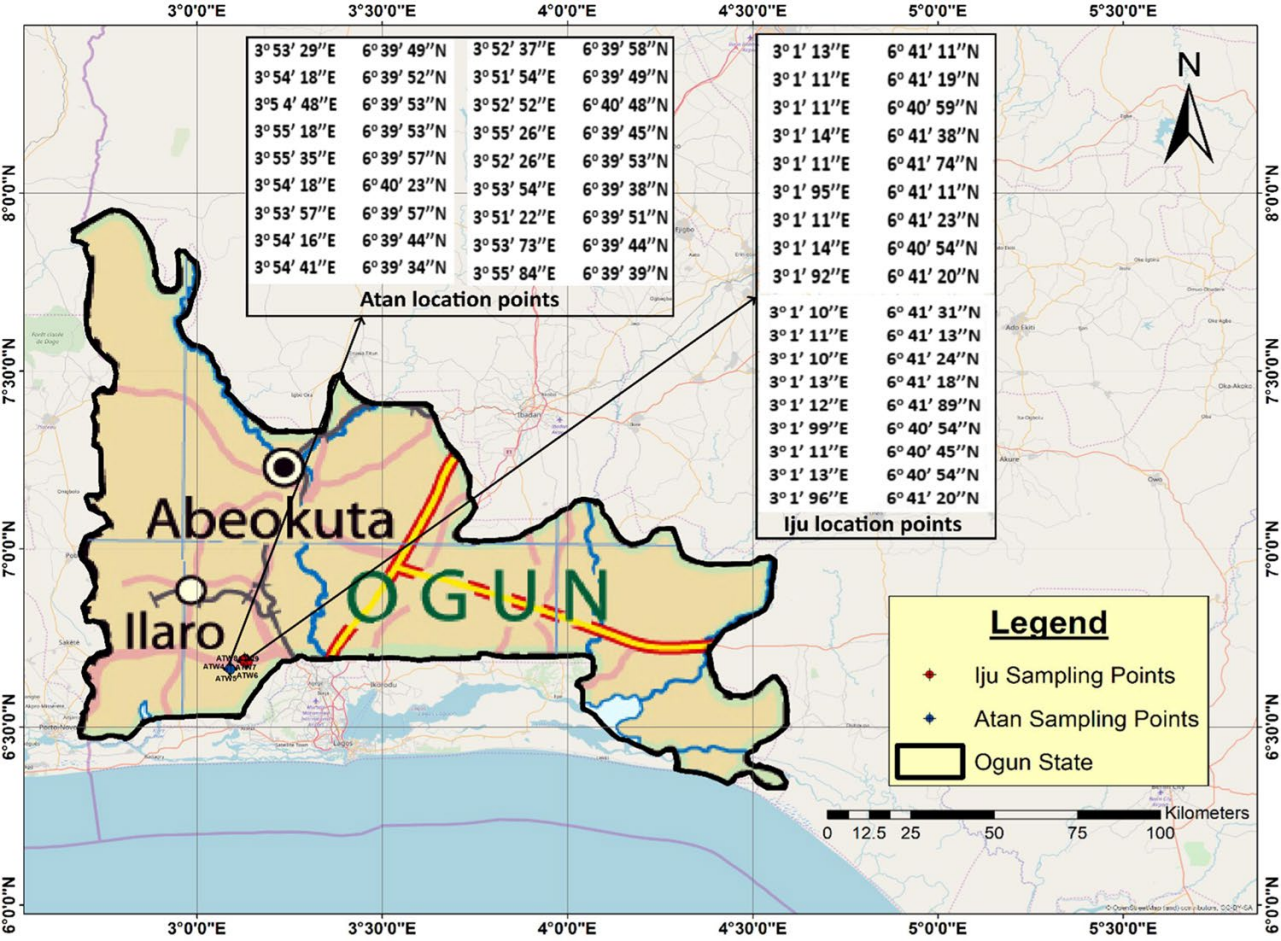
Study area map showing geographic locations of sampling spots.

Iju and Atan are frontline communities situated in Ado-Odo Ota local government area, Ogun state in Southwestern Nigeria (Fig. 1). Atan is located at latitude 6° 46′ 0″ *N* and longitude 2° 48′ 0″ *E*, whereas Iju is situated between 6° 40′ 0″ *N* and longitude 3° 08′ 0″ *E*. Both communities lie at the connective route of Nigeria and the Republic of Benin (62 km northeast of Cotonou), and also links directly to the suburb of Sango-Ota township. Both communities are brimming with approximately 146, 000 inhabitants, with a growth rate of 3.5% annually^59^. The geological characteristics of the region consists of partly complex rock basement of pre-Cambrian formation and partly sedimentary formation, spreading throughout the southwestern part of Nigeria. The vegetation attributes consist of swampy (mangrove and edaphic trees), rainforest and derived forest types^60,61^. For proper analytical reflections, sampling was targeted at locations where water usage is consistent, and the population counts are high.

### Sample collection

This study sampled water from boreholes and hand-dug wells that are regularly used by dwellers for drinking and other domestic activities. In total, 108 water samples from 36 locations were analysed. Standard methods for the analysis of water were adopted and necessary precautions were observed. The groundwater sampling protocol outlined by Plazinska *et al*.^62^ was strictly followed in the collection of water samples from hand-dug wells and boreholes. For each borehole, water was allowed to run for 30 mins before sampling. Whereas in the case of hand-dug wells, dwellers were allowed to collect as much water as they would require before samples were obtained with a water sampler. The reason is to ensure that samples collected represent the actual quality of the aquifer (as in the case of boreholes) and that from the casing (as in the case of wells).

Samples collected were transferred to a 100 cl high-density polyethylene (HDPE) bottle. Before this, the HDPE bottles had been washed with phosphate-free detergent, rinsed with 20% HNO_3_, followed by deionised water and air dried before taking it to the site. Furthermore, The HDPE bottles were rinsed with the sample water thrice before representative samples were collected. *In situ* testing were carried out at each sampling point to determine the pH, electrical conductivity (EC), total dissolved solids (TDS) and temperature. It was necessary to measure these water parameters due to their ability to change during transportation. After acquiring the representative samples, a few drops of 5% HNO_3_ was added immediately to prevent biological growth and precipitation of metals. Samples were labelled appropriately, placed in an icebox and transported to the laboratory where they were stored in a refrigerator (regulated at 4°C) for further analysis. The heavy metals analysed in the present work were selected in line with the observations of different investigators within nearby towns and regions^63–65^. Sensitive water parameters were measured using a waterproof multiparameter meter (HANNA edge EC/Salinity/TDS meter HI2030). Heavy metal concentration in the samples was analysed with an atomic absorption spectrophotometer AAS (model AA – 6800) attached to a graphite furnace atomiser (model GFA–EX7) and an autosampler (model ASC – 6100). For each sample, triplicate measurements were carried out, and recalibration was done after analysing nine water samples.

### Data analysis

After the laboratory analyses, descriptive statistics and Pearson’s Correlation analyses were performed using TIBCO Software Inc. Statistica (data analysis software system), version 13. Furthermore, XLSTAT 2018.5 was used to reduce the dimensionality of the dataset by performing principal factor analysis (PFA). The PFA (Factor loading, scree plot, boxplot, influence plots and loading plots) identifies HM intrusion into the water sources by generating new sets of variables called principal factors (PF). Geospatial analysis (spatial distributions) of HMs considered in this study was performed using ArcMap 10.3.1. Inverse distance weighting (IDW), a type of interpolation technique, was used to create independent raster layers linked with HMs at different points within the study region. This technique has been adopted by investigators to conduct environmental monitoring, understand groundwater chemistry, and to predict pollutant spread^20,66–68^. The principle of the IDW technique centres on data extraction from a subpopulation of the original dataset to develop independent trends.

### Quality control

Quality assurance was realized through the application of standard laboratory measures and quality control methods which included replication, standardized adjustments, use of analytical grade chemical blanks and spikes, and following standard operational procedures. Multiparameter instruments were calibrated each time samples are to be taken from each location. Triplicate analyses were adopted throughout, and the mean value recorded per sample. Throughout the metal concentration measurements, standard detection boundaries were observed, and reagent blanks were monitored throughout the analytical process.

### Human health risk analysis

Several researchers have adopted the health risk assessment model proposed by the US EPA,^69^ to extensively evaluate the potential risks posed by hazardous chemical substances on human health. In the study, HRA of heavy metal concentration was assessed considering two significant pathways through which dwellers are exposed (dermal and ingestion). Ingestion is the most common route, but since the water sources are utilised for other domestic activities, we also considered the dermal route. With respect to the behavioural and physiological variations of different population categories, the exposure risks for adults and children were estimated differently.

Some heavy metals are potential carcinogens while some tend to cause health deterioration when the exposure rates are high. Therefore, we evaluated the carcinogenic and non-carcinogenic risks of specified heavy metal exposure in relation to the allowable threshold concentration outlined by US EPA. To determine the non-carcinogenic risk for ingestion and dermal pathways, the hazard quotients (*HQ*), which is the ratio of the chronic daily intake (*CDI*) of individual metals to the oral reference dose (*RfD*) was calculated using Eq. (1) and Eq. (2) respectively.

1 $$H{Q}_{ingestion}=\frac{CD{I}_{ingestion}}{Rf{D}_{i}}$$

2 $$H{Q}_{dermal}=\frac{CD{I}_{dermal}}{Rf{D}_{d}}$$

Where *HQ*_*Ingestion*_ and *HQ*_*dermal*_ represents the hazard proportion via ingestion and dermal exposure pathways respectively, *CDI*_*Ingestion*_ is the chronic daily intake via ingestion route, whereas *CDI*_*dermal*_ is the chronic daily intake via dermal route. *RfD*_*i*_ and *RfD*_*d*_ are the ingestion and dermal reference dose of HMs respectively. The values of *RfD* adopted for individual metals are as follows; 1.0 × 10^3^ (*μg*/*kg*•*day*) for Al, 1.5 × 10^3^ (*μg*/*kg*•*day*) for Cr, 7 × 10^2^ for Fe, 1.4 × 10^2^ (*μg*/*kg*•*day*) for Mn and 2.0 × 10^1^ (*μg*/*kg*•*day*) for Ni^70^, and 1.4 × 10^2^ (*μg*/*kg*•*day*) for Pb^71^.

The *CDI* of HMs via ingestion and dermal routes were estimated in line with US EPA,^70^ recommendation and the expressions are represented in Eq. (3) and Eq. (4) respectively.

3 $$CD{I}_{ingestion}=\frac{{C}_{HMW}\times I{R}_{W}\times E{F}_{r}\times ED}{BW\times A{T}_{r}}$$

4 $$CD{I}_{dermal}=\frac{{C}_{HMW}\times SA\times {K}_{p}\times E{F}_{r}\times ED\times ET\times CF}{BW\times A{T}_{r}}$$

*C*_*HMw*_ is the concentration of HM in water (*μg*/*L*); *SA* represents the exposed skin area (*cm*^2^); *IR*_*w*_ is the intake rate (*L*/*day*); *K*_*p*_ stands for the coefficient of dermal permeability for water (*unitless*); *ED* is the exposure duration (*year*); *CF* is the unit conversion factor (*L*/*cm*^3^); *ET* is the water exposure time (*hours*/*day*); *EF*_*r*_ is the resident exposure frequency (*days*/*year*); *BW* is body weight (*kg*) and *AT*_*r*_ is the averaging resident time (*days*/*year*).

In the case of carcinogenic risk assessment, we calculated the lifetime cancer risk (*LTCR*) which can be defined as the likelihood of a person developing cancer throughout a lifetime due to the exposure of a specific metal or mixture of metals. The expression for computing *LTCR* is outlined in Eq. (5).

5 $$LTCR=CD{I}_{ingestion}\times SF$$

where *CDI*_*ingestion*_ is the chronic daily intake of heavy metal through the ingestion pathway and *SF*_*HM*_ is the oral slope factor of heavy metal. The slope factor of HMs considered in this study are as follows; 1.7 (*mgkg*^−1^*day*^−1^) for Ni^72^, 0.0085 (*mgkg*^−1^*day*^−1^) for Pb, and 0.5 (*mgkg*^−1^*day*^−1^) for Cr (extracted from the California Toxicity Criteria database and the Integrated Risk Information System database respectively). The acceptable standard of *LTCR* values ranges from 10^−6^ to 10^−4 ^^31^. Therefore, any value above 10^−4^ is considered inadequate or abnormal and could result in cancer over a lifetime. Furthermore, Companion by Minitab (version 5.2.0.0) was used to perform the MCS with 10, 000 iterations. Two population categories (adult and children) were considered, and the results of the simulated exposures for adult and children were expressed at 90^th^, 95^th^, 99^th^, and 99.9^th^ percentile. The reference value for computing *CDI*_*dermal*_ and *CDI*_*ingestion*_ are listed in the supplementary material (Table S1).

## Results and Discussion

### Physicochemical and HM characteristics of groundwater sources

Explanatory analysis of the physicochemical parameters of groundwater samples examined in the study is summarised in Table S2a,b. The parameters analysed include aluminium (Al), chromium (Cr), cadmium (Cd), copper (Cu), iron (Fe), manganese (Mn), nickel (Ni), lead (Pb), pH, total dissolved solids (TDS), electrical conductivity (EC), calcium (Ca) and magnesium (Mg), and their concentrations were compared to World Health Organization (WHO) standard^73^ and the Nigerian Standard for Drinking Water Quality (NSDWQ)^74^. The ratio of samples that exceeded the stipulated limits to the total number of samples analysed was used to calculate the percentage violation at each location.

Considering the HM analysed in all groundwater samples in the study region, Cu and Cd were not detected. Cr was not identified at Atan district but was present in well and borehole samples obtained from Iju district. From Table S2a, the pH value varied from 5.25 to 7.65 (Mean ± SD; 6.67 ± 0.577) at Atan district and 2.59 to 7.23 (5.21 ± 1.299) at Iju district. It is imperative to mention that although pH has no direct impact on humans, its value influences the solubility of metals and the overall geochemistry of groundwater. According to the results, 100% of the well water samples obtained at Iju location violated the WHO standard for pH of drinking water while 55.6% of the borehole samples were above the limits. At Atan location, 33.33% and 77.78% of well-water and borehole samples were within the WHO and NSDWQ pH limits respectively. Woo and Choi^75^ reported that the dissolution of HM from ores could occur at lower pH. Therefore, the low pH observed at Iju location predicts the identification of HM at the region.

The mean values of EC observed at Atan (0.2059 ± 0.0627 µScm^−1^) and Iju (0.7422 ± 0.3012 µScm^−1^) respectively were below the WHO standard. TDS concentration varied from 163.48 to 773.43 mgL^−1^ (mean value of 440.056 ± 187.55 mgL^−1^) and 152.81 to 782.91 mgL^−1^ (mean value of 451.419 ± 221.42 mgL^−1^) at Atan and Iju district respectively. According to Chabukdhara *et al*.^11^, increase in TDS could be as a result of fertiliser application, rainwater application, and sediment dissolution. It is important to mention that dwellers within the study region engage in intensive vegetable farming which encourages the application of fertilizers. Despite the disparity in the TDS concentration, WHO standard was not exceeded and no TDS limit has be provided in the NSDWQ report.

HM concentration in groundwater samples obtained from Atan district varied from 0.0018 to 0.0394 mgL^−1^ for Fe, 0.001 to 0.0048 mgL^−1^ for Mn, 0.142 to 2.221 mgL^−1^ for Al, 0.0004 to 0.0053 mgL^−1^ for Ni, and 0.0009 to 0.042 mgL^−1^ for Pb. The results also revealed that the WHO threshold for lead in drinking water was exceeded in 44.44% of the borehole samples and 11.13% of the well water samples. Furthermore, WHO and NSDWQ limits for Al in drinking water were exceeded in 94.44% of the total samples collected, while the concentration of Fe, Mn, and Ni in drinking water were within the NSDWQ and WHO threshold.

At Iju district (Table S2b), the HM content in the groundwater samples varied from 0.0473 to 0.2397 mgL^−1^ for Fe, 0.0031 to 0.007 mgL^−1^ for Mn, 2.031 to 6.230 mgL^−1^ for Al, 0.0021 to 0.014 mgL^−1^ for Ni, < 0.00001 to 0.0088 mgL^−1^ for Cr and 0.0117 to 0.4272 mgL^−1^ for Pb. In all of these, 100% of the samples exceeded the NSDWQ and WHO standard values for Pb and Al in drinking water, while the concentration of Ni, Cr, Fe, and Mn in the water samples were within the tolerable limits set by the WHO.

Other important water parameters considered are Ca and Mg, which are indicators of water hardness. Ca and Mg concentration varied from 26.144 to 50.433 mgL^−1^ (mean value of 42.328 mgL^−1^) and 55.086 to 98.143 mgL^−1^ (mean value of 76.461 mgL^−1^) respectively at Atan location. Calcium concentration in all the water samples at Atan location were within the WHO permissible guide. However, 5.56% of the water samples surpassed the WHO most desirable limit (MDL) of 50 mgL^−1^ for Ca but none exceeded the NSDWQ threshold. Furthermore, 100% of the water samples exceeded the WHO MDL and NSDWQ of Mg of 50 mgL^−1^ and 20 mgL^−1^ respectively in drinking water. At Iju district, Mg and Ca concentration ranged from 45.025 to 90.231 mgL^−1^ (average value of 66.344 mgL^−1^) and 56.734 to 219.287 mgL^−1^ (average value of 126.301 mgL^−1^). The results also revealed that 27.78% of the water samples were within the WHO MDL for Ca content in drinking water and 100% of the samples surpassed the WHO MDL for Mg in drinking water. With respect to the NSDWQ, 44.44% of the samples surpassed the limit of Ca in drinking, while 100% of the samples exceeded the threshold of Mg in drinking water. The variation identified could be as a result of the dissolution of soil and rock constituents in the water. Also, in as much as Fe content in the water samples were within the WHO guidelines, Magesh *et al*.^67^ reported that ionic interactions in aquifer could result in the presence of Fe in the aquifer.

### Statistical assessment

#### Correlation analysis (CA)

The chemical characteristics of groundwater can be affected by water recharge, parent rock constituents, and residence time of groundwater the aquifer. However, the degree of participation, level of influence and relationship between water quality parameters can be understood by conducting correlation analysis (CA). The CA of the different water quality parameters at each district produces a correlation matrix that demonstrates the relationship between each parameter. However, to describe the strength of a relationship between the parameters, we adopted a correlation coefficient less than 0.5 as weak, a correlation coefficient ranging from 0.5 to 0.6999 as moderate, and a correlation coefficient of ≥ 0.7 as strong. Table S3(a) in the supplementary material summarises the correlation coefficients of water quality parameters at Atan district. From the results, a strong positive association exist between Fe^+^ and Ni (*r* = 0.9125), Al and Ni (*r* = 0.9253), Ni and Ca (*r* = 0.8492), Al and Mn (*r* = 0.8080), Al and Fe (*r* = 0.8083), Al and Ca (*r* = 0.8701), Ca and Mn (*r* = 0.7449) and a moderate positive correlation was observed between Ni and TDS (*r* = 0.6738), Ni and Mn (*r* = 0.6875), TDS and Fe (*r* = 0.6900), Ca and Fe (*r* = 0.6908), TDS and Ca (*r* = 0.6113), TDS and Al (*r* = 0.6913). It is worth mentioning that a strong positive correlation existing between multi-contaminants can be attributed to a common source with which verification can be achieved using principal factor analysis (PFA).

At Iju district, a strong positive correlation was observed between Fe and Pb (*r* = 0.9593), Ca and Cr (*r* = 0.9156), Al and Cr (*r* = 0.8686), TDS and Cr (*r* = 0.8495), Ca and Al (*r* = 0.8917), Mg and Al (*r* = 0.8489), TDS and Ca (*r* = 0.8407), Ca and Mg (*r* = 0.8568), Mg and Cr (*r* = 0.8885), Mg and Pb (*r* = 0.8222), Cr and Pb (*r* = 0.7863), Al and Pb (*r* = 0.7580), EC and Pb (*r* = 0.7777), Al and TDS (*r* = 0.7252), Mg and TDS (*r* = 0.7939) (Table S3b). A positive relationship between TDS, Pb, Cr, Al, EC, Ca and Mg suggests that the salinity of the water sources within the district are affected by the aforementioned water quality parameters. Furthermore, moderate positive correlation was observed between Pb and TDS (*r* = 0.6785), Pb and Ca (*r* = 0.6409), Pb and Mn (*r* = 0.5313), Cr and Fe (*r* = 0.6288), Cr and EC (*r* = 0.5589), Al and Fe (*r* = 0.6046), EC and Fe (*r* = 0.6990), Mg and EC (*r* = 0.6029). In contrast, a strong negative relationship was observed between pH and Cr (*r* = −0.7102), pH and TDS (*r* = −0.7614), pH and Ca (*r* = −0.7723), while a moderate negative correlation was recorded between Mg and pH (*r* = −0.6581), pH and Al (*r* *=* −0.5414). The variation in inter-constituent relationship could be attributed to ion exchange and redox reaction occurring within the groundwater aquifer.

#### Pollution source identification

Validation of inter-elemental relationship as well as pin-pointing pollutant source was achieved using the PFA tool. The PFA tool decomposes stacked dataset into eigenvalues which in turn provides direction within sample observations characterized by high variability. The analysed variability is linked to the correlated variables to obtain principal factors (PF) that are linearly independent. The PF which serves as a representative of the original variables, are regarded as single observations, with the highest variability in the observations outlined in the first PF. Other variations unaccounted for in the first PF will, therefore, be listed in the succeeding components.

**Figure 2:**
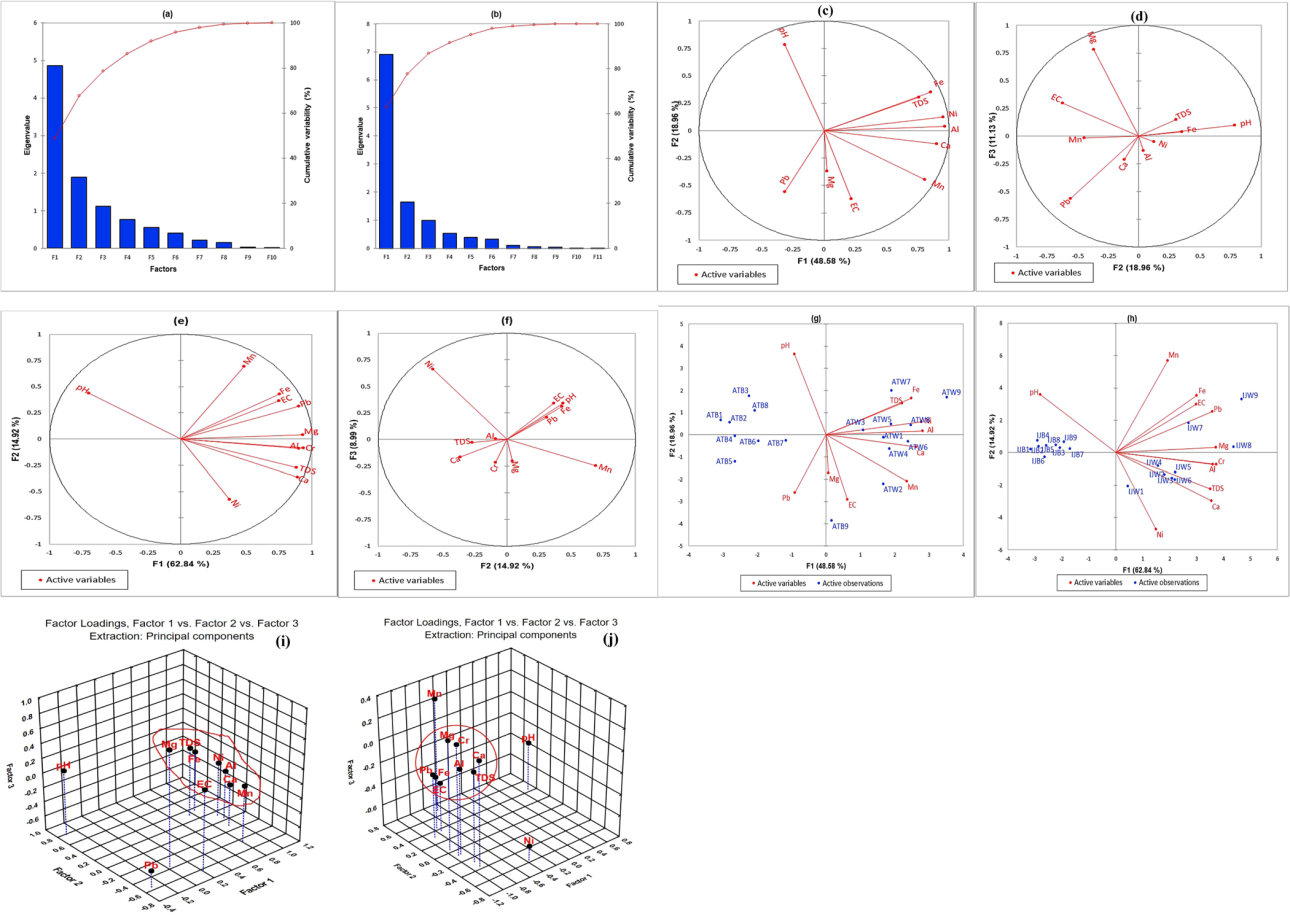
Scree plot of principal components (**a**) Atan (**b**) Iju; Factor loading plot of HMs at Atan location showing (**c**) Factor 1 vs. Factor 2 (**d**) Factor 2 vs. Factor 3 and at Iju location showing (**e**) Factor 1 vs. Factor 2 (**f**) Factor 2 vs. Factor 3; Biplot of HMs distribution and location groupings at (g) Atan considering Factor 1 vs. Factor 2 and (**h**) Iju considering Factor 1 vs. Factor 2; 3D plot of Factor loadings showing Factor 1 vs. Factor 2 vs. Factor 3 (**i**) Atan (**j**) Iju.

The results of the PFA produced a scree plot that identified three significant components within the two districts that account for 78.674% (Atan district) and 86.753% (Iju district) of the data variability respectively (Fig. 2(a,b)). The components of PF1 and PF2 at Atan district explained 67.54% of the data variability with PF1 contributing 48.58% and PF2 contributing 18.96% of the variability respectively. The combined contributions at PF1 produced a cluster of high positive variables which includes Fe, TDS, Ni, Al, Ca and Mn, whereas PF2 recorded high positive contribution from pH only (Table S4(a), Fig. 2(c,d)). EC showed high negative loadings in PF2, while Pb showed a slightly moderate negative loading in PF2 Considering the percentage contribution of the aforementioned variables, Al (19.212%) recorded the highest, followed by Ni, Ca, Fe, Mn and TDS with corresponding values of 18.765%, 16.696%, 14.994%, 13.438% and 11.879% respectively. Furthermore, the percentage contribution from pH towards PF2 was observed to be 32.305% (See Table S4b). It can be suggested that PF1 identifies contamination from both natural and anthropogenic activities. Meanwhile, the presence of TDS, Al, Fe, Mn and Ca affirms the impact of natural processes such as geological metamorphosis and soil/rock weathering (evaporite, feldspar, schistose). In addition, metals like Fe and Mn could be linked to anthropogenic actions such as electric power generation from fossil fuel, welding, heating and refrigeration, paper mills and power plants. Meanwhile, the presence of Ni suggests emissions from metal industries, pipe fittings and power plants. Confirmation of the presence of these industries have been outline by Adewumi *et al*.^76^. However, it is also important to note that geodetic sources such as clay minerals and sandstone could leach Ni into groundwater sources. Nickel content in water is a function of the parent rock formation that makes up the aquifer structure as well as anthropogenic activities. The study region sits on a cretaceous deposition of Palaeocene age, mottled clay, shale, and Palaeocene Ostracods fauna. However, these formations are laden with different Ni concentrations^77,78^. In line with this, combined anthropogenic and geodetic intrusions might be responsible for the presence of Ni in the groundwater sources^79^. In addition to the source-location identification, the variable cluster of PF1 at Atan district get their contributions from well-water sources such as ATW9 (14.139%), ATW8 (6.977%), ATW6 (6.563%), ATW7 (4.144%) and ATW4 (3.883%) (Table S4(e) and Fig. 2g). However, the highest contributors to the variable cluster at PF2 include ATB9 (43.564%) and ATW2 (14.227%). The well-water sources within the aforementioned location were located close to metallurgy production and vehicle painting industry.

Similarly, the outcome of the PFA conducted at Iju district revealed that two PF (PF1 and PF2) accounted for 77.76% of the total variation. Therefore, major explanation will focus on PF1 and PF2. PF1 contributed 62.84% while PF2 added 14.92% of the total variance (Fig. 2b). PF1 is characterized by significant positive variables such as Cr (*r* *=* 0.936), Mg (*r* *=* 0.931), Al (*r* *=* 0.900), Pb (*r* *=* 0.899), Ca (*r* *=* 0.889), TDS (*r* *=* 0.880), Fe (*r* *=* 0.753) and EC (*r* *=* 0.746) (See Table S4(c), Fig. 2(e,f)). Meanwhile, PF2 recorded moderate positive contributions from Mn (*r* *=* 0.692), and a weak positive input from pH (*r* *=* 0.439). Moderate negative loading was observed for Ni (−0.576) and a weak negative loading was recorded for TDS and Ca (−0.273 and −0.363 respectively).

The order of contributions of various water quality constituents to PF1 can be ranked in decreasing form of Cr < Mg < Al < Pb < Ca < TDS < Fe < EC with corresponding values of 12.677%, 12.550%, 11.729%, 11.684%, 11.444%, 11.203%, 8.202% and 8.058% respectively (Table S4d). Considering PF2, Mn contributed 29.211%, while pH added 11.713% to the entire variability. The cluster characterised by Cr, Al, Pb, TDS, Fe and EC suggest anthropogenic sources mainly from industrial activities such as tanneries, galvanising, textiles, pesticides, foundries located in Iju district. Also, the significant negative contribution of Ni (20.195%) signifies that its presence originated from a unique source. To identify the location with the influence attributes mentioned above, the biplot (Fig. 2h) revealed that the well-water sources located at IJW9, IJW8 and IJW7 are laden with high level of Pb, Mg, EC and Fe. Other locations influenced by Al, Cr, TDS and Ca include IJW4, IJW2 IJW3, IJW5 and IJW6. To illustrate the combined influence of the first three components at both districts, PF1, PF2 and PF3 were plotted in a 3D graph (Fig. 2(i,j))

Comparing the outcome of this study and other works in literature, the mean value of Pb concentration obtained from Atan district were less than average values reported by Chabukdhara *et al*.^11^ in Ghaziabad, India; Sirmaur district, India^1^, Costa Rica^80^; Al-Qassim in Saudi Arabia^81^; Durpur and Jallah Jeen in Pakistan^82^, and Pasir Mas in Malaysia^83^, but higher than the mean values of Pb concentration obtained from Guangxi Zhuang region in China^84^, Shanxi province in China^85^. However, the mean concentration of Pb recorded at Iju were higher than the Pb concentration obtained in the works mentioned above.

Considering the mean concentration of Cr obtained in this study, the values recorded at Atan and Iju District were less than the mean values obtained at Durpur and Jallah Jeen in Pakistan^82^, East Singhbhum in India^49^, and Sirmaur district in India^1^. However, the mean values obtained at Iju district were higher than the mean concentration gotten from Yazd Province, Iran^20^, and Southern Peninsular, India^67^.

### Mechanisms of heavy metal toxicity

Heavy metals have the ability to replace some cations in water resulting in health toxicity. Lead tends to replace bivalent cations such as Mg^2+^, Ca^2+^ and Fe^2+^ which in turn, causes harm to the biological mechanisms of cells. The ionic interaction of Pb in water affects cells adhesion, ionic transportation, protein folding, apoptosis, and cellular signalling^86^. Protein kinase C which is responsible for memory storage and the regulation of neural excitation is denatured after interaction with Ca^2+^^87^. Aluminium disrupts most cellular and physical processes. However, adequate period of Al toxicity has not been allocated because its symptoms can be detected at several intervals after aluminium exposure^88^. Aluminium when consumed, replaces Fe^3+^ and Mg^2+^ in the body. The exchange results in several instabilities related with cellular growth, intercellular interaction and secretory functions. These complications share similar degenerative lesions symptoms seen in Alzheimer patients^89^. Iron causes several disruptions in the human body. For example, when Fe^2+^ fails to bind with proteins in the body, it forms free radicals affecting iron levels in biological fluids and mammalian cells. If ingested in high concentration, Fe^2+^ becomes corrosive and affects the gastrointestinal tract. Other complications include penetration into the cells of the liver, heart and brain^86^. According to Albretsen^90^, free iron obtained from excessive intake can result in lipid peroxidation which causes damage to the mitochondria and microsomes. Similarly, Grazuleviciene *et al*.^91^ reported that the hydrogen-free-radicals derived from excessive absorption of iron are responsible for cellular damage, malignant alterations and mutation. Although the health implication of the presence of the identified HMs were not ascertained in the current study, the heightened concentration provides insight on the health toxicity that may arise from ingesting water considering the sources investigated.

### Spatial distribution

**Figure 3:**
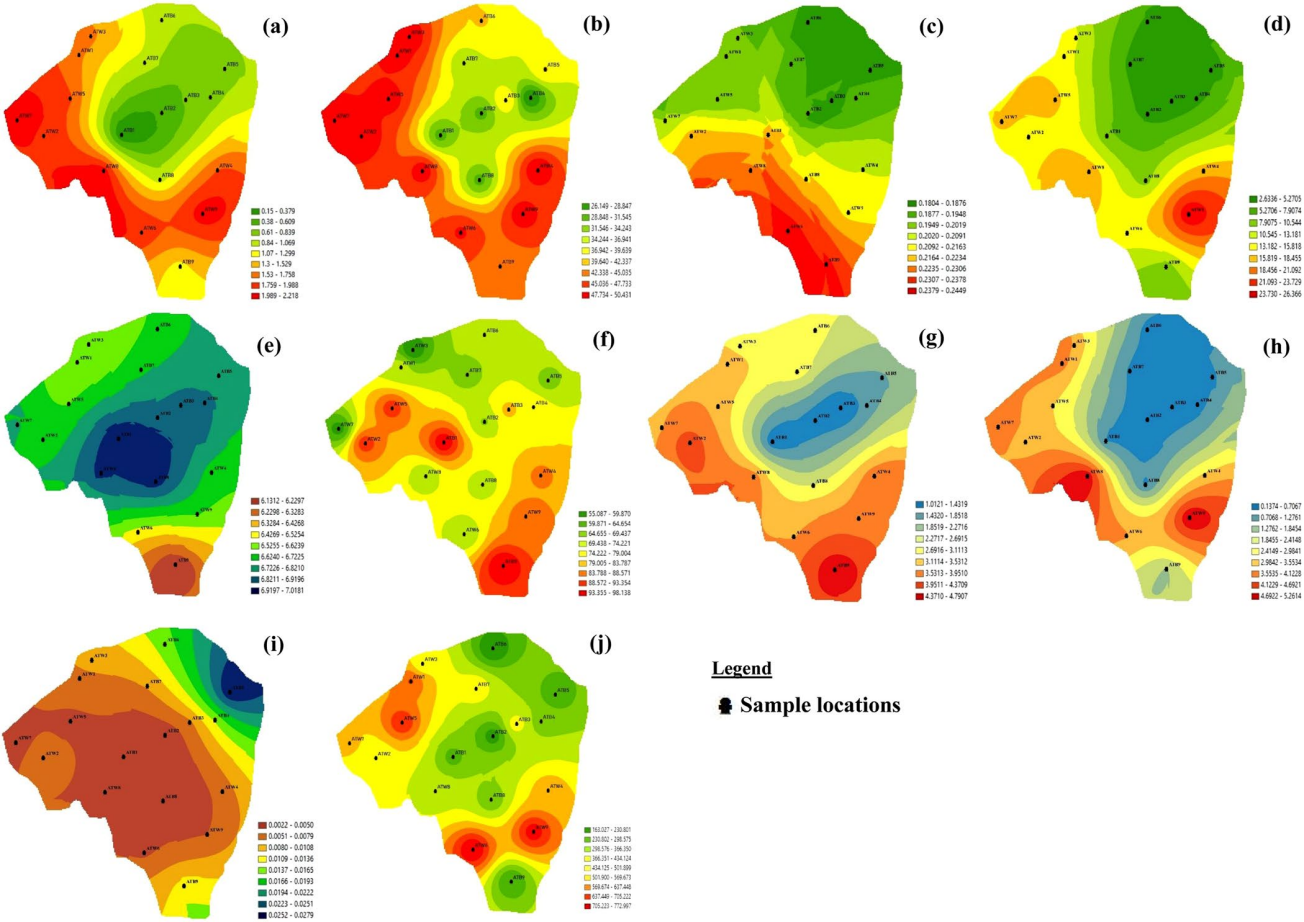
Spatial distribution of water parameters considered in Atan Location (**a**) Al (**b**) Ca (**c**) EC (**d**) Fe (**e**) Mg (**f**) Mn (**g**) Ni (**h**) Pb (**i**) pH (**j**) TDS.

**Figure 4:**
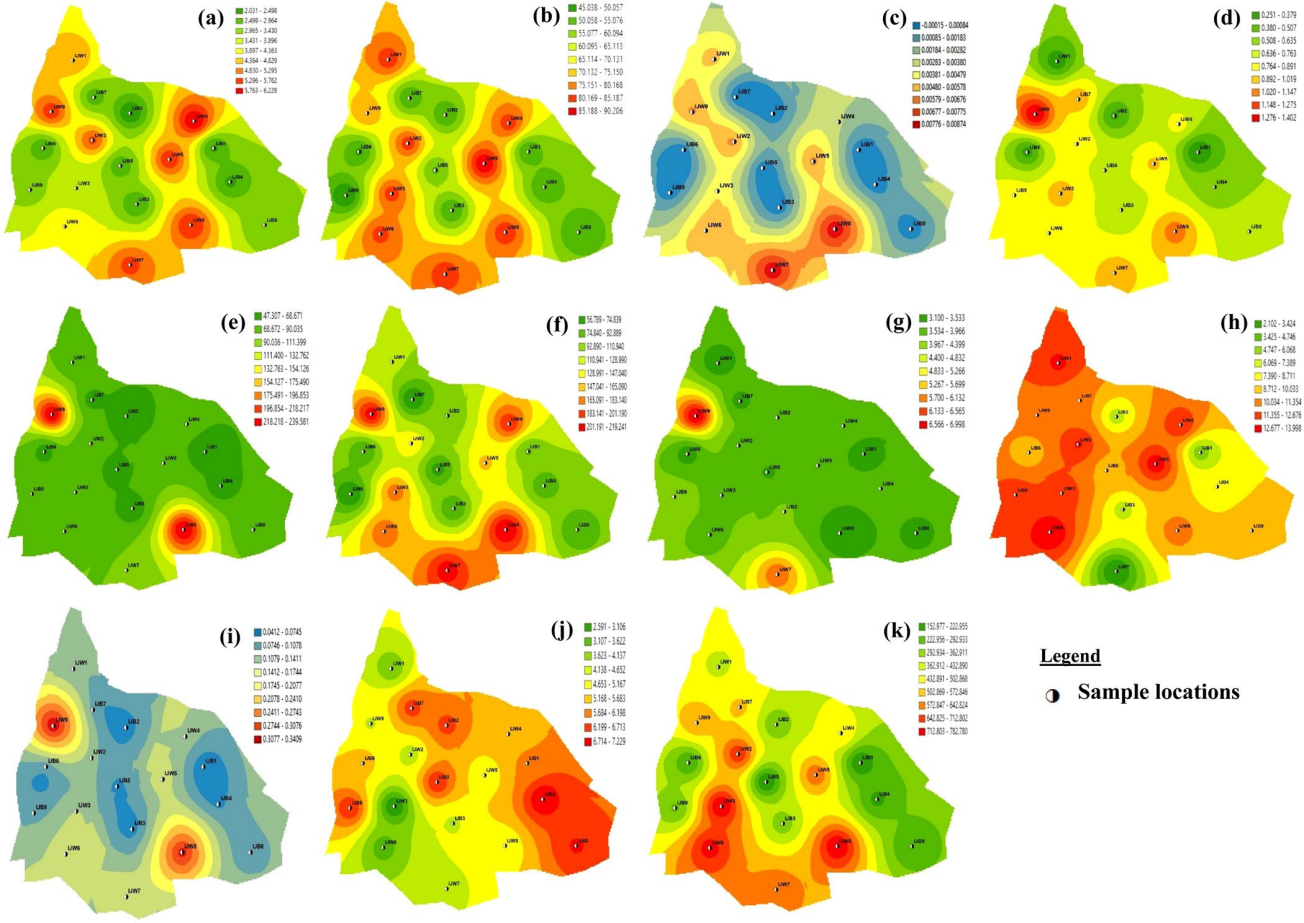
Spatial distribution of water parameters considered in Iju Location (**a**) Al (**b**) Ca (**c**) Cr (**d**) EC (**e**) Fe (**f**) Mg (**g**) Mn (**h**) Ni (**i**) Pb (**j**) pH (**k**) TDS.

Figure 3 and Fig. 4 represents the spatial distribution maps of the individual water parameters and heavy metals considered in Atan and Iju district respectively. The spatial analysis for each parameter was plotted separately for each studied district using the IDW technique.

### Health risk assessment

#### Ingestion risk

Computing the exposure risk of metals through different medium is of great importance as it helps to study the toxicity level of water sources to human health. A number of metals were investigated in the study, but detailed attention was given to Pb. It is noteworthy to mention that the *HQ*_*ingestion*_ values of Al, Fe, Mn, and Ni in the borehole samples and Al, Fe, Mn, Cr, and Ni in the well-water samples at Iju district were below the acceptable standard set by the US EPA. However, the *HQ*_*ingestion*_ computed for Pb for both sources at Iju district is of great concern and worthy of attention. The probability health risk associated with the metals analysed from the different water sources in this study are highlighted in Table 1. The computed *HQ*_*ingestion*_ values identified higher risk associated with Pb at Iju location where the Pb *HQ*_*ingestion*_ values were above the permissible level for both adult and children (*HQ*_*ingestion*_ >1). For adult, the mean value of *HQ*_*ingestion*_ for borehole water was 1.108, while the 90^th^, 95^th^, 99^th^, and 99.9^th^ percentile were equivalent to 1.753, 1.938, 2.296 and 2.632 respectively (Table 1).

**Table 1:** Ingestion probabilistic health risk for boreholes and water wells in Iju and Atan.

HQ (Adult)							HQ (Child)					
**Ingestion probabilistic health risk for boreholes locations in Iju**												
**Metals**	**Mean**	**SD**	**90th Percentile**	**95th Percentile**	**99th Percentile**	**99.9th Percentile**	**Mean**	**SD**	**90th Percentile**	**95th Percentile**	**99th Percentile**	**99.9th Percentile**
Al	7.792E-02	1.030E-02	9.121E-02	9.502E-02	1.017E-01	1.102E-01	1.317E-01	1.743E-02	1.540E-01	1.605E-01	1.723E-01	1.851E-01
Cd	ND	ND	ND	ND	ND	ND	ND	ND	ND	ND	ND	ND
Fe	5.948E-03	9.070E-04	7.119E-03	7.438E-03	8.019E-03	8.767E-03	1.002E-02	1.542E-03	1.199E-02	1.254E-02	1.368E-02	1.498E-02
Pb	1.108E + 00	5.040E-01	1.753E + 00	1.938E + 00	2.296E + 00	2.632E + 00	1.864E + 00	8.389E-01	2.948E + 00	3.240E + 00	3.828E + 00	4.528E + 00
Cu	ND	ND	ND	ND	ND	ND	ND	ND	ND	ND	ND	ND
Mn	5.433E-03	4.500E-04	6.007E-03	6.174E-03	6.476E-03	6.793E-03	9.158E-03	7.600E-04	1.013E-02	1.040E-02	1.092E-02	1.153E-02
Cr	ND	ND	ND	ND	ND	ND	ND	ND	ND	ND	ND	ND
Ni	1.282E-02	3.157E-03	1.689E-02	1.804E-02	2.015E-02	2.312E-02	2.171E-02	5.357E-03	2.860E-02	3.044E-02	3.427E-02	3.738E-02
**Ingestion probabilistic health risk for water wells locations in Iju**												
Al	1.117E-01	9.990E-03	1.244E-01	1.280E-01	1.352E-01	1.434E-01	1.887E-01	1.652E-02	2.098E-01	2.159E-01	2.273E-01	2.428E-01
Cd	ND	ND	ND	ND	ND	ND	ND	ND	ND	ND	ND	ND
Fe	1.160E-02	7.011E-03	2.057E-02	2.313E-02	2.817E-02	3.336E-02	1.960E-02	1.187E-02	3.496E-02	3.925E-02	4.701E-02	5.578E-02
Pb	4.559E + 00	2.499E + 00	7.750E + 00	8.624E + 00	1.028E + 01	1.239E + 01	7.606E + 00	4.200E + 00	1.297E + 01	1.443E + 01	1.708E + 01	2.104E + 01
Cu	ND	ND	ND	ND	ND	ND	ND	ND	ND	ND	ND	ND
Mn	6.362E-03	1.989E-03	8.934E-03	9.630E-03	1.097E-02	1.250E-02	1.077E-02	3.348E-03	1.508E-02	1.625E-02	1.857E-02	2.103E-02
Cr	6.057E-02	1.813E-02	8.384E-02	9.049E-02	1.031E-01	1.156E-01	1.024E-01	3.068E-02	1.421E-01	1.529E-01	1.736E-01	1.994E-01
Ni	1.694E-02	5.401E-03	2.375E-02	2.592E-02	2.971E-02	3.444E-02	2.850E-02	9.103E-03	4.008E-02	4.357E-02	4.958E-02	5.683E-02
**Ingestion probabilistic health risk for boreholes locations in Atan**												
Al	2.220E-02	9.650E-03	3.472E-02	3.814E-02	4.472E-02	5.341E-02	3.720E-02	1.641E-02	5.818E-02	6.426E-02	7.562E-02	8.837E-02
Cd	ND	ND	ND	ND	ND	ND	ND	ND	ND	ND	ND	ND
Fe	3.130E-04	1.220E-04	4.690E-04	5.130E-04	5.970E-04	6.840E-04	5.290E-04	2.100E-04	7.980E-04	8.800E-04	1.020E-03	1.171E-03
Pb	2.347E-01	2.849E-01	5.978E-01	7.061E-01	8.924E-01	1.098E + 00	4.088E-01	4.646E-01	1.010E + 00	1.182E + 00	1.485E + 00	1.830E + 00
Cu	ND	ND	ND	ND	ND	ND	ND	ND	ND	ND	ND	ND
Mn	3.349E-03	1.837E-03	5.702E-03	6.413E-03	7.723E-03	9.186E-03	5.655E-03	3.085E-03	9.595E-03	1.072E-02	1.288E-02	1.487E-02
Cr	ND	ND	ND	ND	ND	ND	ND	ND	ND	ND	ND	ND
Ni	1.018E-03	6.460E-04	1.842E-03	2.091E-03	2.534E-03	2.991E-03	1.728E-03	1.094E-03	3.122E-03	3.515E-03	4.268E-03	5.141E-03
**Ingestion probabilistic health risk for water wells locations in Atan**												
Al	5.682E-02	7.146E-03	6.588E-02	6.863E-02	7.361E-02	7.951E-02	9.591E-02	1.197E-02	1.114E-01	1.154E-01	1.236E-01	1.328E-01
Cd	ND	ND	ND	ND	ND	ND	ND	ND	ND	ND	ND	ND
Fe	1.968E-03	8.550E-04	3.056E-03	3.364E-03	3.955E-03	4.659E-03	3.293E-03	1.450E-03	5.161E-03	5.698E-03	6.664E-03	7.765E-03
Pb	1.049E-01	7.969E-02	2.061E-01	2.363E-01	2.935E-01	3.594E-01	1.755E-01	1.326E-01	3.434E-01	3.903E-01	4.786E-01	5.916E-01
Cu	ND	ND	ND	ND	ND	ND	ND	ND	ND	ND	ND	ND
Mn	5.391E-03	5.490E-04	6.079E-03	6.297E-03	6.703E-03	7.108E-03	9.096E-03	9.060E-04	1.025E-02	1.059E-02	1.120E-02	1.193E-02
Cr	ND	ND	ND	ND	ND	ND	ND	ND	ND	ND	ND	ND
Ni	5.806E-03	1.292E-03	7.455E-03	7.929E-03	8.836E-03	9.922E-03	9.756E-03	2.157E-03	1.251E-02	1.329E-02	1.492E-02	1.640E-02

**Figure 5:**
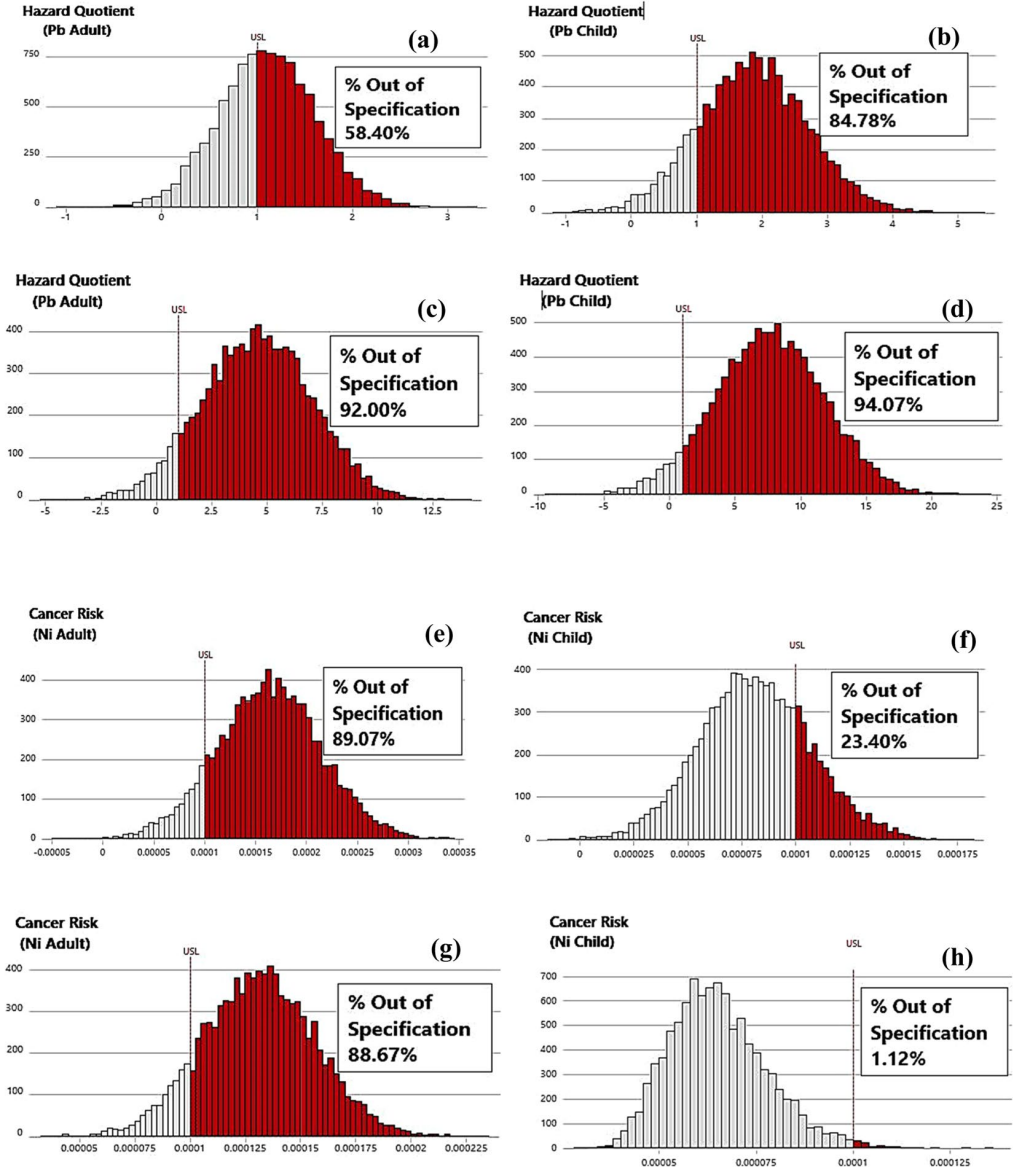
Predicted HQ_*ingestion*_ associated with Pb in Borehole water for (**a**) adult (**b**) child at Iju district, and well-water for (**c**) adult (**d**) child at Iju district Predicted HQ_*ingestion*_ associated with Ni in Borehole water for (**e**) adult (**f**) child at Iju district, and well-water for (**g**) adult (**h**) child at Iju district.

Further analysis using Monte Carlo simulation revealed that despite the *HQ*_*ingestion*_ of borehole water being greater than 1, only 58.40% of the simulated values were greater than the US EPA specification (Fig. 5a). For children, the mean value of *HQ*_*ingestion*_ for borehole water was 1.864, while the 90^th^, 95^th^, 99^th^, and 99.9^th^ percentile were equal to 2.948, 3.240, 3.828, and 4.528 respectively. However, the results indicated that 84.78% of the simulated values surpassed the acceptable limit (Fig. 5b)

Considering the *HQ*_*ingestion*_ of well-water samples at Iju district, the average value of 4.599 and 7.606 were obtained for adult and children respectively. At 90^th^, 95^th^, 99^th^, and 99.9^th^ percentile, the *HQ*_*ingestion*_ values obtained were 7.750, 8.624, 10.028 and 12.390 respectively for adult, and *HQ*_*ingestion*_ values of 12.970, 14.43, 17.08 and 21.04 respectively for children. Further observation showed that 92.00% and 94.07% of the simulated *HQ*_*ingestion*_ values were above the allowable limit for adult and children respectively (Fig. 5c,d).

In the case of Atan district, the health hazard associated with Al, Fe, Pb, Mn, and Ni were considerably below the allowable limit (less than 1) for both water sources considered in this study. For adults and children, considering borehole consumption, the *HQ*_*ingestion*_ values at 50^th^ percentile for Pb ingestion were 2.34 × 10^−1^ and 4.088 × 10^−1^ respectively. In a similar vein, the *HQ*_*ingestion*_ for well-water at 50^th^ percentile were 1.049 × 10^−1^ for adult, and 1.755 × 10^−1^ for children. Other inference reveals the absence of health deficiencies to water users at Atan district in relation to the sources investigated in this study. However, the outcome of the investigation extracted from water data at Iju district identifies the need for stringent waste disposal technique and a robust waste management policy for the industries located within the region. These measures are necessary owing to the life-threatening consequences posed by the long-term usage of borehole water and well-water sources within the region. In addition, a potential lead threat has been identified towards adults and children as a result of continuous usage of water from the aforementioned sources. Invariably, children are susceptible to detrimental health consequences, and may not detoxify the exposure by reason of their under-developed body structure. In comparison, the *HQ*_*ingestion*_ values of Pb at the 50^th^ percentile obtained in the current study were lesser than the values obtained at Igun-Ijesha, Osun State Nigeria^92^, drinking water sources of Dadinkowa dam in Gombe State, Nigeria^93^, water sources consumed in Balogun, Ogun State Nigeria^58^, and water sources of a pipeline vandalised region in Lagos State Nigeria^94^. Furthermore, *HQ*_*ingestion*_ values obtained in the current were higher than the values obtained at water sources of Benin^95^, and surface water sources consumed around Ilesha^96^.

#### Dermal risk

Table S5,6 shows the dermal probabilistic health risk associated to the HM intake from different sources. The mean *HQ*_*dermal*_ values obtained from borehole and well-water samples at Atan and Iju district were less than the tolerable limit. Therefore, no potential health implication (considering adult and children) is associated with the exposure of HM in water as a result of dermal contact. Furthermore, the *HQ*_*dermal*_ values of all metals considered in this study were below 1 at all levels (50^th^, 90^th^, 95^th^, 99^th^, and 99.9^th^ percentile). This suggests that there is no significant cancer risk that would arise from the use of the groundwater in the study areas for bathing^97^. Reported that the total hazard index (ingestion and dermal contact) of heavy metals in groundwater sampled in Malwa Region of Punjab were below or near 1, which indicated non-carcinogenic health risk from the use of the water. Similar studies have shown that higher carcinogenic and non-carcinogenic risks can be attributed to ingestion pathways^98,99^

#### Lifetime cancer risk

The prediction of potential lifetime cancer risk (*LTCR*) for Pb, Ni and Cr were studied at both districts. For well-water sources at Iju district, the average *LTCR*_*Child*_ for Pb, Ni, and Cr are 8.157 × 10^−7^, 8.10 × 10^−5^, and 1.30 × 10^−5^ respectively. The 5^th^ and 95^th^ percentile obtained were 7.00 × 10^−6^ and 2.10 × 10^−5^ for Cr; 3.90 × 10^−5^ and 1.24 × 10^−4^ for Ni; 1.547 × 10^−7^ and 1.00 × 10^−6^ for Pb. Similarly, the calculated average *LTCR*_*Adult*_ for Cr, Ni, and Pb are 2.70 × 10^−5^, 1.63 × 10^−4^ and 2.00 × 10^−6^ respectively. The 5% and 95% risk were obtained as 1.50 × 10^−5^ and 4.20 × 10^−5^ for Cr; 7.90 × 10^−5^ and 2.48 × 10^−4^ for Ni; 7.378 × 10^−7^ and 3.00 × 10^−6^ for Pb (Table 2). Comparing the risk obtained in this study to the maximum allowable risk (1 × 10^−4^), 89.07% of the Nickel LTCR values for adults fell outside of the maximum allowable standard and 23.40% of the Nickel cancer risk values for children exceeded the permissible limits (Fig. 5e,f). These results suggest that cancer risk is higher in adults than in children in the study areas. This is contrary to the findings of Taiyuan, China where it was reported that cancer risk due to ingestion was higher in children than in adults^100^.

**Table 2:** LTCR associated with HMs in well-water and borehole water in Iju and Atan.

LTCR Child				LTCR ADULT		
**LTCR associated with HMs in well-water at Iju**						
**Metals**	**Mean**	**5** ^**th**^ **Percentile**	**95** ^**th**^ **Percentile**	**Mean**	**5** ^**th**^ **Percentile**	**95** ^**th**^ **Percentile**
Cr	0.000013	0.000007	0.000021	0.000027	0.000015	0.000042
Ni	0.000081	0.000039	0.000124	0.000163	0.000079	0.000248
Pb	8.157E-7	1.547E-7	0.000001	0.000002	7.378E-7	0.000003
LTCR associated with HMs in borehole water at Iju						
Cr	ND	ND	ND	ND	ND	ND
Ni	0.000065	0.000047	0.000088	0.000131	0.000089	0.000173
Pb	0.000002	9.847E-7	0.000003	0.000004	0.000002	0.000006
LTCR associated with HMs in well-water at Atan						
Cr	ND	ND	ND	ND	ND	ND
Ni	0.000028	0.00002	0.000039	0.000056	0.000039	0.000078
Pb	1.626E-7	−4.457E-8	3.675E-7	3.289E-7	−9.263E-8	7.484E-7
LTCR associated with HMs in borehole water at Iju						
Cr	ND	ND	ND	ND	ND	ND
Ni	0.000005	0.000002	0.000009	0.00001	0.000004	0.000018
Pb	4.09E-7	−3.252E-7	0.000001	8.13E-7	−6.488E-7	0.000002

In the case of borehole water sources at Iju, the LTCR values for Cr was not modelled as the concentration was below the detection limit. However, the average cancer risk for Ni was 6.50 × 10^−5^ and 1.37 × 10^−4^ for children and adult respectively. The mean cancer risk calculated for Pb equalled to 2.00 × 10^−6^ for children, and 4.00 × 10^−6^ for adult. Further analysis revealed that 1.12% of the Nickel *LTCR* values for children surpassed the specification limits, while 88.67% of the cancer risk values for adult were above the stipulated standard (Fig. 5g,h).

LTCR analysis conducted for the HMs in well-water samples from Atan location indicated that the mean LTCR for Ni was 2.80 × 10^−5^ for children and 5.60 × 10^−5^ for adult. LTCR for Pb recorded a mean value of 1.626 × 10^−7^ for children and 3.289 × 10^−7^ for adult. However, these values were below the maximum permissible standard by the WHO. Similar scenario was observed for borehole water samples as the LTCR values were found to be below the stipulated standard (See Table 2).

An investigation by Koki *et al*.^53^ revealed that the LTCR for adults were higher when matched with children after long-term exposure to the groundwater in Klang valley and Melaka, Malaysia. Fallahzadeh *et al*.^20^ reported high carcinogenic risk for lead in groundwater samples in Ardakan, and a high risk for Nickel in groundwater samples in Meibod and Bahabad.

#### Sensitivity analysis

This study also performed sensitivity analysis to understand how input variables contribute to the probabilistic cancer risk assessment. The results indicated positive influential contribution from the input variables in order of Concentration > EF > IR > ED for lead and Nickel in Atan. In comparison, the impact of the variables on the risk estimation was prominent in Nickel than lead. However, BW had a negative impact on the LTCR calculation for both metals in Atan district (See Fig. S1(a,b). At Iju, the sensitivity analysis revealed concentration as the highest contributor (40.7% and 53.3% for lead and nickel respectively), followed by IR (15.1% and 12.2% for lead and nickel respectively), AT (6.8% and 5.6% for lead and nickel respectively), EF (2.2% and 3.8% for lead and nickel respectively), and a negative influence from BW (−34.2% and −25.1% for lead and nickel respectively) (Fig. S1(c,d) in supplementary file).

Regardless of the results obtained from the water quality analysis considering the few water parameters analysed and low number of samples collected, this study reveals a fraction of the environmental risk inhabitants are constantly exposed to. Therefore, the current study should be taken as preliminary.

#### Uncertainty analysis

In the present study, Monte Carlo Simulation (MCS) approach adopted entails the input of random range of values for each parameter in the estimation of the exposure risk at each site. The population group considered were obtained through a large number of simulations (10, 000) in order to derive predictive quantities as the output. Estimating potential health risk in this regard, depends on distribution-based approach rather than deterministic risk estimation approach (point-based calculation) to obtain the probabilistic output at each site. Therefore, the MCS technique integrates variabilities and uncertainties in the risk calculations. However, for accurate estimation of the risk assessment (in the field and during simulation), several uncertainties were considered. First, the sampling points investigated were selected in the region of high population density to describe the study region. Second, accuracy of HM concentration assessment was achieved by taking into consideration, several precautionary measures associated with sample extraction, transportation and storage, quality assurance and control steps reported in earlier sections, and the use of standardized equipment in the laboratory. Third, the input and output parameters were quantified mathematically using probabilistic distributions which signifies an array of risks experienced by several members of the population group^101^. The MCS technique offers a holistic quantitative approach of evaluating the probabilistic distribution within the boundaries of the estimation model for health risk assessment and exposure^102^.

## Conclusion

Physicochemical and HM concentration in groundwater, geospatial variations, pollution sources and health risk assessment were examined within Iju and Atan district, South-western Nigeria. Based on the water quality results of the 108 samples analysed from both location, 100% of the well-water and 55.6% of borehole samples at Iju exceeded the WHO threshold for pH in water. Meanwhile, at Atan district, the pH violation recorded for well-water and borehole samples were 66.7% and 22.22% respectively. For both locations, lead content in the groundwater sources was relatively high. The analytical outcome revealed that 100% of well-water samples and borehole samples violated the WHO guidelines at Iju district. Whereas, Atan district recorded a 44.44% and 11.13% Pb violation in borehole and well-water samples respectively. In addition, water quality parameters such as Mg and Ca surpassed the WHO tolerable limits at both locations. Source identification of metals were related to geogenic and anthropogenic intrusions. The inter-parameter relationship extracted from the CA and PFA techniques revealed that the geogenic contributions were as a result of dissolutions, weathering, geological metamorphosis and leaching. However, anthropogenic contributions emanated from industrial (galvanising, foundries, textile production, tanneries) activities with the region. A further observation from the non-carcinogenic risk analysis identified potential threats from Pb via ingestion routes for both children and adult living within Iju district. The potential threats identified were related to both groundwater sources considered in the study, and in specific terms, the Monte Carlo simulations pinpointed that 58.40% of the adult and 84.78% of the children population are at risk on ingesting borehole water. The results also indicated that ingesting Pb-contaminated well-water exposes 92% of the adult and 94.07% of the children population to life-threatening health scenarios. It is also necessary to mention that no potential threats from Pb via dermal routes were identified considering both sources and location. However, carcinogenic risk was identified for Ni in borehole and well-water sources within Iju.

Generally, the outcome of water analysis and health risk assessment conducted within the region, with regards to the non-carcinogenic risk posed by Pb ingestion, revealed that the risks are higher for children compared to adult. But, in the case of Ni carcinogenic risk, the risks were higher for adults compared to children. Therefore, this study calls for a robust water management strategy that incorporates policy implementation, recurrent water monitoring programmes, proper wastewater disposal strategy, resource accountability and effective water and wastewater treatment, so as to safeguard the lives of inhabitants within the study region.

## Supplementary information

Supplementary Dataset 1
