# Supplementary material for: Probabilistic risk assessment and spatial distribution of potentially toxic elements in groundwater sources in Southwestern Nigeria
Source: Sci Rep. 2019 Nov 4;9:15920. doi: 10.1038/s41598-019-52325-z (PMC6828721; doi:10.1038/s41598-019-52325-z)
Supplement: Supplementary file 1 — Supplementary Dataset 1 [file 41598_2019_52325_MOESM1_ESM.docx]

**Probabilistic risk assessment and spatial distribution of potentially toxic elements in groundwater sources in Southwestern Nigeria**

**Order of authors**

1. Emenike, PraiseGod Chidozie *

[*praisegod.emenike@covenantuniversity.edu.ng*](mailto:praisegod.emenike@covenantuniversity.edu.ng)

Department of Civil Engineering,

Covenant University, Ota, Ogun State, Nigeria

*(Corresponding author)

+2348036051722

1. Tenebe, Imokhai

*[itt1@txstate.edu](mailto:itt1@txstate.edu)*

Ingram School of Engineering,

Texas State University, San Marcos, Texas, USA.

1. Ogarekpe, Nkpa

[*nkpaogarekpe@yahoo.com*](mailto:nkpaogarekpe@yahoo.com)

Department of Civil Engineering,

Cross River University of Technology, Calabar, Nigeria

1. Omole, David

[*david.omole@covenantuniversity.edu.ng*](mailto:david.omole@covenantuniversity.edu.ng)

Department of Civil Engineering,

Covenant University, Ota, Ogun State, Nigeria

1. Nnaji, Chidozie^a,b^
   [*chidozie.nnaji@unn.edu.ng*](mailto:chidozie.nnaji@unn.edu.ng)
   **a.** Department of Civil Engineering,
   University of Nigeria, Nsukka, Enugu State, Nigeria

**b.** Faculty of Engineering and Built Environment

University of Johannesburg,

South Africa

**Supplementary Material**

Table S1: Key parameters for computing the exposure risk of heavy metals through oral and dermal pathways

| **Variables** | **Unit** | **Distribution** | **Min** | **Average** | **Max** | **Std Dev** | **Ref** |
| --- | --- | --- | --- | --- | --- | --- | --- |
| Concentration of HM | mg/L | Derived from data | - | - | - | - | Our study |
| Intake Rate (Water) – Adult | L/day | Triangular | 0.9 | 2.5 | 9.5 | - | - |
| Intake Rate (Water) – Child | L/day | Triangular | 0.1 | 0.71 | 5.5 | - | - |
| Exposure duration (ED) | year | Lognormal | 11.36 | - | - | 13.72 | USDOE ^1^ |
| Exposure Frequency (EF) | Day/year | Triangular | 180 | 345 | 365 | - | USDOE ^1^ |
| Body Weight (BW) – Child | kg | Triangular | 6.5 | 15 | 26.1 |  | Binkowitz and Wartenberg ^2^ |
| Body Weight (BW) – Adult | kg | Lognormal | 76.71 | - | - | 11.19 | Binkowitz and Wartenberg ^2^ |
| Averaging Time (AT) – Child | Day | Fixed | 2190 | - | - | - | USDOE ^1^ |
| Averaging Time (AT) – Adult | Day | Fixed | 8760 | - | - | - | USDOE ^1^ |
| Skin Surface Area (SA) - Child | cm^2^/day | Triangular | 430 | 830 | 2160 | - | Wang et al. ^3^ |
| Skin Surface Area (SA) - Adult | cm^2^/day | Triangular | 760 | 1530 | 4220 | - | USDOE ^1^ |
| Dermal permeability (K) | cm/hour | Fixed | 0.001 | - | - | - | Baqar et al. ^4^ |

Table S2(a): Descriptive statistics of analyzed water samples at Atan (N^d^ = 54 from 18 different locations)

|  | **Pb (mg/L)** | **Ni (mg/L)** | **Fe (mg/L)** | **Mn (mg/L)** | **Al (mg/L)** | **pH** | **EC (µScm^–1^)** | **TDS (mg/L)** | **Ca (mg/L)** | **Mg (mg/L)** |
| --- | --- | --- | --- | --- | --- | --- | --- | --- | --- | --- |
| **Mean** | 0.0080 | 0.0023 | 0.0114 | 0.0029 | 1.3188 | 6.6711 | 0.2059 | 440.0556 | 42.3281 | 76.4612 |
| **StDev** | 0.0098 | 0.0017 | 0.0104 | 0.0011 | 0.6571 | 0.5769 | 0.0627 | 187.5471 | 8.4180 | 12.6905 |
| **Range** | 0.0411 | 0.0049 | 0.038 | 0.0038 | 2.0792 | 2.4000 | 0.2060 | 609.9800 | 24.2897 | 43.0569 |
| **Minimum** | 0.0009 | 0.0004 | 0.0018 | 0.001 | 0.1417 | 5.2500 | 0.1060 | 163.4800 | 26.1437 | 55.0859 |
| **Maximum** | 0.0420 | 0.0053 | 0.0394 | 0.0048 | 2.2209 | 7.6500 | 0.3120 | 773.4600 | 50.4333 | 98.1428 |
| **Q1** | 0.0021 | 0.00058 | 0.0027 | 0.0018 | 0.7094 | 6.3200 | 0.1475 | 268.0875 | 33.9883 | 68.6291 |
| **Q2** | 0.0043 | 0.0024 | 0.0075 | 0.0032 | 1.3990 | 6.6200 | 0.2135 | 445.4700 | 46.1606 | 71.4690 |
| **Q3** | 0.0110 | 0.00367 | 0.0180 | 0.0037 | 1.9122 | 7.1075 | 0.2498 | 567.0325 | 49.5028 | 87.9811 |
| **95.0% CI Mean** | 0.003 to 0.013 | 0.0014 to 0.0032 | 0.0062 to 0.0166 | 0.0024 to 0.0035 | 0.992 to 1.646 | 6.384 to 6.958 | 0.175 to 0.237 | 346.790 to 533.320 | 38.142 to 46.514 | 70.150 to 82.772 |
| **95.0% CI Sigma** | 0.007 to 0.015 | 0.0013 to 0.0027 | 0.0078 to 0.0156 | 0.0008 to 0.0017 | 0.493 to 0.985 | 0.433 to 0.865 | 0.047 to 0.094 | 140.730 to 281.160 | 6.317 to 12.620 | 9.523 to 19.025 |
| **Skewness** | 2.6936 | 0.3137 | 1.2432 | -0.4356 | -0.1770 | -0.3619 | -0.0041 | 0.3162 | -0.6648 | 0.2650 |
| **Kurtosis** | 8.6751 | -1.4253 | 1.5394 | -0.7998 | -1.3129 | 1.0394 | -1.0789 | -0.8748 | -1.1038 | -0.8032 |

Table S2(b): Descriptive statistics of analyzed water samples at Iju (N^d^ = 54 from 18 different locations)

|  | **Pb (mg/L)** | **Cr (mg/L)** | **Ni (mg/L)** | **Fe (mg/L)** | **Mn (mg/L)** | **Al (mg/L)** | **pH** | **EC (µScm^–1^)** | **TDS (mg/L)** | **Ca (mg/L)** | **Mg (mg/L)** |
| --- | --- | --- | --- | --- | --- | --- | --- | --- | --- | --- | --- |
| **Mean** | 0.1320 | 0.0030 | 0.0099 | 0.0881 | 0.0039 | 3.9062 | 5.2078 | 0.7422 | 451.4189 | 66.3440 | 126.3007 |
| **StDev** | 0.1160 | 0.0034 | 0.00320 | 0.0566 | 0.00098 | 1.4714 | 1.2993 | 0.3012 | 221.4156 | 17.1874 | 59.1457 |
| **Range** | 0.4155 | 0.0088 | 0.0119 | 0.1924 | 0.0039 | 4.1991 | 4.6400 | 1.1530 | 630.1000 | 45.2054 | 162.5532 |
| **Minimum** | 0.0117 | ND | 0.0021 | 0.0473 | 0.0031 | 2.0309 | 2.5900 | 0.2500 | 152.8100 | 45.0252 | 56.7340 |
| **Maximum** | 0.4272 | 0.0088 | 0.0140 | 0.2397 | 0.0070 | 6.2300 | 7.2300 | 1.4030 | 782.9100 | 90.2306 | 219.2872 |
| **Q1** | 0.0478 | 0.0000 | 0.0074 | 0.0591 | 0.0034 | 2.5313 | 4.4325 | 0.4875 | 226.4950 | 50.0105 | 74.8217 |
| **Q2** | 0.0984 | 0.0016 | 0.0105 | 0.0735 | 0.0037 | 3.3967 | 4.9950 | 0.7880 | 461.6600 | 64.5359 | 102.5428 |
| **Q3** | 0.1751 | 0.0061 | 0.0127 | 0.0880 | 0.00385 | 5.4825 | 6.4825 | 0.9460 | 643.3850 | 82.9239 | 182.7822 |
| **95.0% CI Mean** | 0.074304 to 0.18966 | 0.0013684 to 0.0047205 | 0.0084 to 0.0115 | 0.0599 to 0.1162 | 0.00345 to 0.00443 | 3.1745 to 4.6379 | 4.5616 to 5.8539 | 0.5924 to 0.89193 | 341.31 to 561.53 | 57.797 to 74.891 | 96.888 to 155.71 |
| **95.0% CI Sigma** | 0.087036 to 0.17388 | 0.002529 to 0.0050526 | 0.0024 to 0.0048 | 0.0425 to 0.0848 | 0.00074 to 0.0015 | 1.1041 to 2.2059 | 0.97499 to 1.9479 | 0.22599 to 0.45149 | 166.15 to 331.93 | 12.897 to 25.766 | 44.382 to 88.668 |
| **Skewness** | 1.657 | 0.431939 | -0.906826 | 2.428 | 2.481 | 0.285491 | -0.209460 | 0.081544626 | 0.139626 | 0.048353691 | 0.395392 |
| **Kurtosis** | 2.468 | -1.455 | 0.483513 | 5.026 | 5.924 | -1.713 | -0.686227 | 0.002026313 | -1.496 | -2.092 | -1.541 |
| **WHO limits** | 0.01 | 0.05 | 0.02 | 0.3 | 0.05 | 0.10 – 0.20 | 6.5 – 8.5 | 1500 | 1000 | MDL = 50 | MDL = 50 |
| **NSDWQ** | 0.01 | 0.05 | 0.02 | 0.3 | 0.2 | 0.2 | 6.5 – 8.5 | - | - | 75 | 20 |

MDL = (Most desirable limit); N^d^ = number of samples; Q1= First quartile; Q2 = Second quartile; Q3 = Third quartile; CI = Confidence Interval; StDev = Standard deviation

Table S3(a): Correlation matrix of HMs at Atan district

|  | Correlations | | | | | | | | | |
| --- | --- | --- | --- | --- | --- | --- | --- | --- | --- | --- |
| Variable | Pb | Ni | Fe | Mn | Al | pH | EC | TDS | Ca | Mg |
| Pb | 1 |  |  |  |  |  |  |  |  |  |
| Ni | -0.3030 | 1 |  |  |  |  |  |  |  |  |
| Fe | -0.3757 | 0.9125 | 1 |  |  |  |  |  |  |  |
| Mn | -0.0592 | 0.6875 | 0.4945 | 1 |  |  |  |  |  |  |
| Al | -0.2617 | 0.9253 | 0.8083 | 0.8080 | 1 |  |  |  |  |  |
| pH | -0.2546 | -0.1484 | 0.0358 | -0.5704 | -0.2400 | 1 |  |  |  |  |
| EC | 0.0701 | 0.1814 | -0.0519 | 0.4037 | 0.1767 | -0.3454 | 1 |  |  |  |
| TDS | -0.4337 | 0.6738 | 0.6900 | 0.3931 | 0.6913 | -0.0612 | 0.0376 | 1 |  |  |
| Ca | -0.0657 | 0.8492 | 0.6908 | 0.7449 | 0.8701 | -0.4021 | 0.0966 | 0.6113 | 1 |  |
| Mg | -0.0560 | -0.0553 | 0.0070 | 0.1530 | -0.1083 | -0.1988 | 0.2091 | 0.0003 | 0.0011 | 1 |

Table S3(b): Correlation matrix of HMs at Iju district

|  | Correlations | | | | | | | | | | |
| --- | --- | --- | --- | --- | --- | --- | --- | --- | --- | --- | --- |
| Variables | Pb | Cr | Ni | Fe | Mn | Al | pH | EC | TDS | Ca | Mg |
| Pb | 1 |  |  |  |  |  |  |  |  |  |  |
| Cr | 0.7863 | 1 |  |  |  |  |  |  |  |  |  |
| Ni | 0.2662 | 0.2203 | 1 |  |  |  |  |  |  |  |  |
| Fe | 0.9593 | 0.6288 | 0.1897 | 1 |  |  |  |  |  |  |  |
| Mn | 0.5313 | 0.3873 | -0.2447 | 0.4671 | 1 |  |  |  |  |  |  |
| Al | 0.7580 | 0.8686 | 0.3994 | 0.6046 | 0.3755 | 1 |  |  |  |  |  |
| pH | -0.4344 | -0.7102 | -0.3410 | -0.2680 | -0.1984 | -0.5414 | 1 |  |  |  |  |
| EC | 0.7777 | 0.5589 | 0.2917 | 0.6990 | 0.5823 | 0.5942 | -0.2710 | 1 |  |  |  |
| TDS | 0.6785 | 0.8495 | 0.4238 | 0.4921 | 0.2209 | 0.7252 | -0.7614 | 0.6562 | 1 |  |  |
| Ca | 0.6409 | 0.9156 | 0.4343 | 0.4393 | 0.2205 | 0.8917 | -0.7723 | 0.4624 | 0.8407 | 1 |  |
| Mg | 0.8222 | 0.8885 | 0.1707 | 0.6743 | 0.4755 | 0.8489 | -0.6581 | 0.6029 | 0.7939 | 0.8568 | 1 |

Table S4(a): Factor loadings of HMs at Atan district

|  | F1 | F2 | F3 |
| --- | --- | --- | --- |
| Pb | -0.312 | -0.557 | -0.564 |
| Ni | 0.955 | 0.124 | -0.051 |
| Fe | 0.853 | 0.352 | 0.039 |
| Mn | 0.808 | -0.448 | -0.017 |
| Al | 0.966 | 0.036 | -0.131 |
| pH | -0.314 | 0.783 | 0.099 |
| EC | 0.216 | -0.622 | 0.296 |
| TDS | 0.760 | 0.305 | 0.152 |
| Ca | 0.901 | -0.119 | -0.209 |
| Mg | 0.026 | -0.370 | 0.780 |

Table S4(b): Percentage Contribution of HMs to each PF at Atan

|  | F1 | F2 | F3 |
| --- | --- | --- | --- |
| Pb | 2.010 | 16.387 | 28.583 |
| Ni | 18.765 | 0.810 | 0.237 |
| Fe | 14.994 | 6.546 | 0.138 |
| Mn | 13.438 | 10.607 | 0.026 |
| Al | 19.212 | 0.068 | 1.546 |
| pH | 2.033 | 32.305 | 0.875 |
| EC | 0.960 | 20.419 | 7.894 |
| TDS | 11.879 | 4.892 | 2.070 |
| Ca | 16.696 | 0.747 | 3.931 |
| Mg | 0.013 | 7.220 | 54.700 |

Table S4(c): Factor loadings of HMs at Iju district

|  | F1 | F2 | F3 |
| --- | --- | --- | --- |
| Pb | 0.899 | 0.311 | 0.208 |
| Cr | 0.936 | -0.088 | -0.218 |
| Ni | 0.373 | -0.576 | 0.660 |
| Fe | 0.753 | 0.429 | 0.311 |
| Mn | 0.481 | 0.692 | -0.248 |
| Al | 0.900 | -0.090 | 0.007 |
| pH | -0.700 | 0.439 | 0.341 |
| EC | 0.746 | 0.365 | 0.342 |
| TDS | 0.880 | -0.273 | -0.031 |
| Ca | 0.889 | -0.363 | -0.167 |
| Mg | 0.931 | 0.041 | -0.205 |

Table S4(d): Percentage Contribution of HMs to each PF at Iju

|  | F1 | F2 | F3 |
| --- | --- | --- | --- |
| Pb | 11.684 | 5.898 | 4.365 |
| Cr | 12.677 | 0.470 | 4.813 |
| Ni | 2.012 | 20.195 | 44.054 |
| Fe | 8.202 | 11.231 | 9.803 |
| Mn | 3.351 | 29.211 | 6.216 |
| Al | 11.729 | 0.489 | 0.005 |
| pH | 7.091 | 11.713 | 11.747 |
| EC | 8.058 | 8.132 | 11.815 |
| TDS | 11.203 | 4.524 | 0.094 |
| Ca | 11.444 | 8.033 | 2.823 |
| Mg | 12.550 | 0.104 | 4.265 |

Table S4(e): Percentage contribution of source location to each PF at Atan

| Atan District | | | | Iju District | | | |
| --- | --- | --- | --- | --- | --- | --- | --- |
| Stations | F1 | F2 | F3 | Stations | F1 | F2 | F3 |
| ATB1 | 10.729 | 1.303 | 25.865 | IJB1 | 8.030 | 0.142 | 7.787 |
| ATB2 | 8.964 | 0.930 | 0.009 | IJB2 | 6.566 | 0.481 | 2.308 |
| ATB3 | 5.703 | 8.905 | 1.075 | IJB3 | 3.472 | 0.303 | 3.246 |
| ATB4 | 8.051 | 0.006 | 1.421 | IJB4 | 6.804 | 1.969 | 0.046 |
| ATB5 | 8.049 | 4.202 | 30.840 | IJB5 | 5.348 | 0.630 | 2.057 |
| ATB6 | 4.443 | 0.244 | 5.926 | IJB6 | 5.615 | 0.262 | 0.234 |
| ATB7 | 1.562 | 0.203 | 0.036 | IJB7 | 2.306 | 0.184 | 7.781 |
| ATB8 | 4.921 | 3.509 | 0.721 | IJB8 | 3.986 | 0.741 | 4.057 |
| ATB9 | 0.031 | 43.564 | 1.875 | IJB9 | 2.977 | 1.555 | 10.531 |
| ATW1 | 3.204 | 0.033 | 0.997 | IJW1 | 0.161 | 14.225 | 1.175 |
| ATW2 | 3.188 | 14.227 | 2.787 | IJW2 | 2.627 | 6.107 | 0.001 |
| ATW3 | 1.355 | 0.142 | 12.412 | IJW3 | 3.515 | 8.548 | 1.023 |
| ATW4 | 3.883 | 1.149 | 1.341 | IJW4 | 1.988 | 2.237 | 0.317 |
| ATW5 | 4.094 | 0.653 | 3.217 | IJW5 | 3.925 | 4.996 | 1.039 |
| ATW6 | 6.563 | 0.277 | 0.387 | IJW6 | 3.859 | 9.189 | 0.017 |
| ATW7 | 4.144 | 11.642 | 8.906 | IJW7 | 5.889 | 11.296 | 48.297 |
| ATW8 | 6.977 | 0.573 | 0.173 | IJW8 | 15.373 | 0.448 | 6.237 |
| ATW9 | 14.139 | 8.440 | 2.011 | IJW9 | 17.561 | 36.688 | 3.847 |

Table S5(a): Dermal probabilistic health risk for boreholes locations in Atan

| **HQ (Adult)** | | | | | | | **HQ (Child)** | | | | | |
| --- | --- | --- | --- | --- | --- | --- | --- | --- | --- | --- | --- | --- |
| **Metals** | **Mean** | **SD** | **90th Percentile** | **95th Percentile** | **99th Percentile** | **99.9th Percentile** | **Mean** | **SD** | **90th Percentile** | **95th Percentile** | **99th Percentile** | **99.9th Percentile** |
| Al | 1.240E-04 | 5.400E-05 | 1.930E-04 | 2.120E-04 | 2.500E-04 | 2.880E-04 | 1.650E-04 | 2.580E-04 | 2.840E-04 | 3.340E-04 | 3.550E-04 | 2.580E-04 |
| Cd | ND | ND | ND | ND | ND | ND | ND | ND | ND | ND | ND | ND |
| Fe | 1.200E-05 | 5.000E-06 | 1.800E-05 | 1.900E-05 | 2.300E-05 | 2.600E-05 | 1.600E-05 | 6.000E-06 | 2.300E-05 | 2.600E-05 | 3.000E-05 | 3.400E-05 |
| Pb | 4.452E-03 | 5.166E-03 | 1.096E-02 | 1.299E-02 | 1.666E-02 | 2.116E-02 | 5.923E-03 | 6.905E-03 | 1.465E-02 | 1.713E-02 | 2.190E-02 | 2.729E-02 |
| Cu | ND | ND | ND | ND | ND | ND | ND | ND | ND | ND | ND | ND |
| Mn | 4.620E-04 | 2.560E-04 | 7.870E-04 | 8.810E-04 | 1.050E-03 | 1.267E-03 | 6.220E-04 | 3.430E-04 | 1.060E-03 | 1.192E-03 | 1.431E-03 | 1.645E-03 |
| Cr | ND | ND | ND | ND | ND | ND | ND | ND | ND | ND | ND | ND |
| Ni | 4.000E-06 | 3.000E-06 | 8.000E-06 | 9.000E-06 | 1.000E-05 | 1.300E-05 | 6.000E-06 | 4.000E-06 | 1.000E-05 | 1.100E-05 | 1.400E-05 | 1.700E-05 |

Table S5(b): Dermal probabilistic health risk for well-water locations in Atan

| **HQ (Adult)** | | | | | | | **HQ (Child)** | | | | | | |
| --- | --- | --- | --- | --- | --- | --- | --- | --- | --- | --- | --- | --- | --- |
| **Metals** | **Mean** | **SD** | **90th Percentile** | **95th Percentile** | **99th Percentile** | **99.9th Percentile** | **Mean** | **SD** | **90th Percentile** | **95th Percentile** | **99th Percentile** | **99.9th Percentile** | |
| Al | 3.180E-04 | 4.000E-05 | 3.690E-04 | 3.840E-04 | 4.100E-04 | 4.440E-04 | 4.240E-04 | 4.920E-04 | 5.130E-04 | 5.490E-04 | 5.610E-04 | 4.920E-04 |  |
| Cd | ND | ND | ND | ND | ND | ND | ND | ND | ND | ND | ND | ND |  |
| Fe | 7.300E-05 | 3.200E-05 | 1.140E-04 | 1.260E-04 | 1.490E-04 | 1.780E-04 | 9.800E-05 | 4.200E-05 | 1.520E-04 | 1.670E-04 | 1.940E-04 | 2.210E-04 |  |
| Pb | 1.974E-03 | 1.453E-03 | 3.844E-03 | 4.417E-03 | 5.325E-03 | 6.309E-03 | 2.598E-03 | 1.948E-03 | 5.140E-03 | 5.846E-03 | 7.001E-03 | 8.360E-03 |  |
| Cu | ND | ND | ND | ND | ND | ND | ND | ND | ND | ND | ND | ND |  |
| Mn | 7.530E-04 | 7.600E-05 | 8.510E-04 | 8.780E-04 | 9.330E-04 | 9.950E-04 | 1.004E-03 | 1.010E-04 | 1.131E-03 | 1.167E-03 | 1.233E-03 | 1.332E-03 |  |
| Cr | ND | ND | ND | ND | ND | ND | ND | ND | ND | ND | ND | ND |  |
| Ni | 2.400E-05 | 5.000E-06 | 3.100E-05 | 3.300E-05 | 3.700E-05 | 4.200E-05 | 3.200E-05 | 7.000E-06 | 4.100E-05 | 4.300E-05 | 4.800E-05 | 5.400E-05 |  |

HQ = Hazard quotient; ND = Not Detected; SD = Standard deviation

Table S6(a): Dermal probabilistic health risk for boreholes locations in Iju

| **HQ (Adult)** | | | | | | | **HQ (Child)** | | | | | |
| --- | --- | --- | --- | --- | --- | --- | --- | --- | --- | --- | --- | --- |
| **Metals** | **Mean** | **SD** | **90th Percentile** | **95th Percentile** | **99th Percentile** | **99.9th Percentile** | **Mean** | **SD** | **90th Percentile** | **95th Percentile** | **99th Percentile** | **99.9th Percentile** |
| Al | 4.360E-04 | 5.800E-05 | 5.110E-04 | 5.310E-04 | 5.680E-04 | 6.160E-04 | 5.810E-04 | 7.700E-05 | 6.810E-04 | 7.080E-04 | 7.610E-04 | 8.190E-04 |
| Cd | ND | ND | ND | ND | ND | ND | ND | ND | ND | ND | ND | ND |
| Fe | 2.210E-04 | 3.400E-05 | 2.640E-04 | 2.770E-04 | 3.000E-04 | 3.230E-04 | 2.950E-04 | 4.500E-05 | 3.530E-04 | 3.690E-04 | 4.000E-04 | 4.360E-04 |
| Pb | 2.038E-02 | 9.305E-03 | 3.223E-02 | 3.564E-02 | 4.187E-02 | 4.804E-02 | 2.721E-02 | 1.233E-02 | 4.285E-02 | 4.753E-02 | 5.622E-02 | 6.763E-02 |
| Cu | ND | ND | ND | ND | ND | ND | ND | ND | ND | ND | ND | ND |
| Mn | 7.570E-04 | 6.300E-05 | 8.380E-04 | 8.600E-04 | 9.020E-04 | 9.460E-04 | 1.008E-03 | 8.300E-05 | 1.115E-03 | 1.144E-03 | 1.202E-03 | 1.275E-03 |
| Cr | ND | ND | ND | ND | ND | ND | ND | ND | ND | ND | ND | ND |
| Ni | 5.300E-05 | 1.300E-05 | 6.900E-05 | 7.400E-05 | 8.300E-05 | 9.400E-05 | 7.100E-05 | 1.700E-05 | 9.300E-05 | 1.000E-04 | 1.120E-04 | 1.230E-04 |

Table S6(b): Dermal probabilistic health risk for water wells locations in Iju

| **HQ (Adult)** | | | | | | | **HQ (Child)** | | | | | |
| --- | --- | --- | --- | --- | --- | --- | --- | --- | --- | --- | --- | --- |
| **Metals** | **Mean** | **SD** | **90th Percentile** | **95th Percentile** | **99th Percentile** | **99.9th Percentile** | **Mean** | **SD** | **90th Percentile** | **95th Percentile** | **99th Percentile** | **99.9th Percentile** |
| Al | 6.240E-04 | 5.500E-05 | 6.940E-04 | 7.130E-04 | 7.550E-04 | 8.010E-04 | 8.320E-04 | 7.300E-05 | 9.260E-04 | 9.510E-04 | 1.000E-03 | 1.056E-03 |
| Cd | ND | ND | ND | ND | ND | ND | ND | ND | ND | ND | ND | ND |
| Fe | 4.350E-04 | 2.600E-04 | 7.680E-04 | 8.660E-04 | 1.040E-03 | 1.285E-03 | 5.690E-04 | 3.420E-04 | 1.008E-03 | 1.130E-03 | 1.394E-03 | 1.619E-03 |
| Pb | 8.442E-02 | 4.695E-02 | 1.442E-01 | 1.625E-01 | 1.932E-01 | 2.289E-01 | 1.133E-01 | 6.139E-02 | 1.913E-01 | 2.142E-01 | 2.530E-01 | 3.088E-01 |
| Cu | ND | ND | ND | ND | ND | ND | ND | ND | ND | ND | ND | ND |
| Mn | 8.920E-04 | 2.770E-04 | 1.249E-03 | 1.355E-03 | 1.534E-03 | 1.727E-03 | 1.187E-03 | 3.640E-04 | 1.649E-03 | 1.784E-03 | 2.041E-03 | 2.322E-03 |
| Cr | 6.779E-02 | 2.028E-02 | 9.399E-02 | 1.011E-01 | 1.142E-01 | 1.302E-01 | 9.057E-02 | 2.712E-02 | 1.252E-01 | 1.354E-01 | 1.535E-01 | 1.782E-01 |
| Ni | 7.000E-05 | 2.300E-05 | 9.900E-05 | 1.070E-04 | 1.210E-04 | 1.400E-04 | 9.300E-05 | 3.000E-05 | 1.310E-04 | 1.420E-04 | 1.630E-04 | 1.840E-04 |

**(b)**

**(a)**

**(d)**

**(c)**

Fig S1: Sensitivity analysis of LTCR model for (a) Nickel (b) lead in Atan, and (c) Nickel (d) Lead in Iju districts

**References**

1. USDOE. *The Risk Assessment Information System. U.S. Department of Energy (DOE), Office of Environmental Management, Oak Ridge Operations (ORO) Office*. (2011).

2. Binkowitz, B. S. & Wartenberg, D. Disparity in Quantitative Risk Assessment: A Review of Input Distributions. *Risk Anal.* **21**, 75–90 (2001).

3. Wang, B., Yu, G., Huang, J. & Hu, H. Development of species sensitivity distributions and estimation of HC 5 of organochlorine pesticides with five statistical approaches. *Ecotoxicology* **17**, 716–724 (2008).

4. Baqar, M. *et al.* Organochlorine pesticides across the tributaries of River Ravi, Pakistan: Human health risk assessment through dermal exposure, ecological risks, source fingerprints and spatio-temporal distribution. *Sci. Total Environ.* **618**, 291–305 (2018).
